# Modelling the impact of different front-of-package nutrition labels on mortality from non-communicable chronic disease

**DOI:** 10.1186/s12966-019-0817-2

**Published:** 2019-07-15

**Authors:** Manon Egnell, Paolo Crosetto, Tania d’Almeida, Emmanuelle Kesse-Guyot, Mathilde Touvier, Bernard Ruffieux, Serge Hercberg, Laurent Muller, Chantal Julia

**Affiliations:** 1 Sorbonne Paris Cité Epidemiology and Statistics Research Centre (CRESS), U1153 Inserm, U1125, Inra, Cnam, University of Paris 13, Nutritional Epidemiology Research Team (EREN), 93017 Bobigny, France; 2 Inra, UMR 1215 GAEL, 38000 Grenoble, France; 3 Polytechnic Institute of Grenoble, 38031 Grenoble, France; 4 Public Health Department, Avicenne Hospital, 93000 Bobigny, France

**Keywords:** Front-of-pack nutrition label, Food labelling, Non-communicable diseases, Consumer behaviour

## Abstract

**Background:**

Front-of-Package nutrition labels (FoPLs) are intended to help reduce the incidence of nutrition-related non-communicable diseases through an improvement in diet quality. FoPLs have been shown to improve the nutritional quality of purchases and have been associated with improved diet quality, which is in turn associated with reduced risk of non-communicable diseases. However, the potential impact of FoPLs on reducing mortality from chronic diseases has never been estimated.

**Methods:**

Data from a laboratory experimental economics test were used to investigate the effects of five different FoPLs (Nutri-Score, Health Star Rating system, Multiple Traffic lights, Reference intakes and SENS (*Système d’Etiquetage Nutritionnel Simplifié*)) on the nutritional quality of household purchases. The relative differences in nutrient content and composition of food purchases were then applied to dietary intakes using data from an observational study, thus yielding estimates for ‘reference’ and ‘labelled’ diets. A macro-simulation study using the PRIME model was then conducted to estimate the impact of the modification in dietary intake as a result of FoPL use on mortality from diet-related non-communicable diseases.

**Results:**

The use of FoPLs led to a substantial reduction in mortality from chronic diseases. Approximately 3.4% of all deaths from diet-related non-communicable diseases was estimated to be avoidable when the Nutri-Score FoPL was used. The remaining FoPLs likewise resulted in mortality reduction, although to a lesser extent: Health Star Rating system (2.8%), Reference Intakes (1.9%), Multiple Traffic Lights (1.6%), and SENS (1.1%).

**Conclusions:**

FoPLs have the potential to help decrease mortality from diet-related non-communicable diseases, and the Nutri-Score appears to be the most efficient among the five formats tested.

**Electronic supplementary material:**

The online version of this article (10.1186/s12966-019-0817-2) contains supplementary material, which is available to authorized users.

## Background

Nutrition-related chronic diseases, including cardiovascular diseases, cancers, and diabetes, have become a major issue for the balance of current healthcare systems [1]. In 2016, 39.5 million deaths from non-communicable diseases were recorded in the world, including 17.6 million from cardiovascular diseases and 8.9 million from cancer [2]. Between 2006 and 2016, worldwide cardiovascular disease and cancer mortality increased by 14.5 and 17.8%, respectively [2]. In France, cardiovascular diseases and cancer cause the majority of deaths, each accounting for about 30% of mortality [3]. For these diseases, one of the major leading risk factors in many countries is poor dietary quality [2]. Given the high disease burden associated with nutrition-related chronic diseases, healthcare authorities have embraced public health policies, aiming at improving diet at the population level in order to reduce risk of nutrition-related diseases. Among the various interventions in this domain, Front-of-Pack nutrition Labels (FoPLs) are receiving growing attention [4]. FoPLs aim at guiding consumer choices towards healthier food products at the point-of-purchase by way of providing simplified, salient and easily understandable information on the nutritional quality of food products [4]. Intervention studies have shown that some FoPLs can significantly improve the nutritional quality of food purchases [5, 6], which may translate into a beneficial impact on dietary intakes. Moreover FoPLs are regarded as incentives for food manufacturers to improve the nutritional quality of their products through reformulations and innovations [7, 8].

The improvement of diets through FoPLs may have a direct impact on the incidence and mortality from nutrition-related chronic diseases, as nutritional intakes are associated with risk of chronic diseases. For example, it has been established that an increase in the consumption of fruit and vegetable is associated with a decreased risk of coronary heart disease [9], and that an increase in fibre intake is associated with a decreased risk of both colorectal cancer and incidence of stroke [10]; in turn, salt intake has been positively associated with blood pressure, which is closely related to risk of stroke and coronary heart disease [11]. Simulation studies assess the overall impact of dietary intake modification on the population level on nutrition-related mortality [12]. Scenarios for modifications in dietary intakes can in particular be generated from studies investigating the effects of specific interventions, and therefore reasonably model their health impacts. Such studies are of importance to policy-makers, as they provide useful estimates of the potential health-related gains from a given intervention [13]. However, even though FoPLs have been described as effective tools for guiding consumer behaviour towards healthier food choices at the point-of-purchase, [5] their potential direct impact on the incidence and mortality from nutrition-related non-communicable diseases (NCDs) remains largely unknown.

Various FoPL formats have been designed around the world. Nutrient-specific labels display information for specific nutrients (fats, Saturated Fatty Acids (SFA), sugars, and salt) using a monochrome (e.g. a modified version of the Reference Intakes) or color-coded format (e.g. the Multiple Traffic Lights, implemented in the United Kingdom in 2005). Summary FoPLs include scale-based graded labels, indicating overall nutritional quality of the product (e.g. the Nutri-Score adopted in France in 2017, or the Health Star Rating system, used in New Zealand and Australia since 2014) or frequency-based labels displaying information on a recommended intake frequency (e.g. the SENS (*Système d’Etiquetage Nutritionnel Simplifié*) label, designed and supported by the French Retail Federation). Several studies have shown that the effects of FoPLs on consumer purchases may vary considerably depending on their graphical format [6, 14]. Their effect on individual diets may also differ (in terms of nutrient intake in particular) which in turn may modulate the effects on health outcomes. Given these considerations, comparing the respective potential impact of different FoPLs on mortality could help guide policy-makers in selecting the most efficient format. However, to the best of our knowledge, no comparative evaluation of the relative effects of different FoPLs formats on dietary intakes is available. Moreover, even if they exist in other countries, FoPLs are implemented in specific geographical and cultural contexts which renders the use of such effect estimates across populations challenging. Therefore, using homogeneous data from a single pool of individuals, and measuring actual purchasing behaviour (as opposed to stated preferences) appears as particularly relevant.

The objective of the present study is to estimate the potential impact of several different FoPLs designs on mortality from chronic diseases in the French population using a macro-simulation model. Estimates of change in dietary intakes were drawn from an experimental frame-field experiment conducted in France prior to the implementation of the Nutri-Score, which compared the following five FoPLs: Nutri-Score, Multiple Traffic Lights (MTL), Reference Intakes (RIs), Health Star Rating (HSR) system, and SENS. The five labels were tested in the same environment using standardised procedures and very similar samples of French participants. In order to test the robustness of our results, scenarios taking into account the variability in consumer responses to the five FoPLs were included in the study.

## Methods

**Fig. 1:**
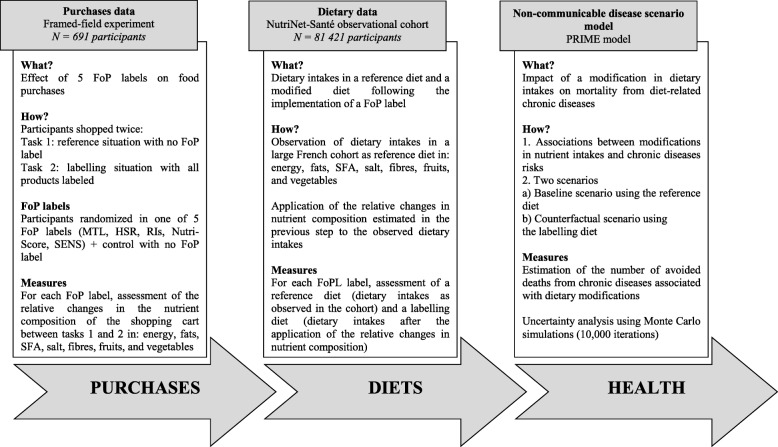
Description of the present study methods

The present study ran a non-communicable disease scenario macro-simulation model to estimate the potential impact of modifications in dietary intakes following the use of a FoPL on pre-packed foods on mortality from NCDs. To run this model, two separate data sources were used: FoPLs effects on the nutrient content and composition of household food purchases were estimated using data from an economics laboratory framed-field experiment, and an observational study was used to assess reference dietary intakes in a large population, using repeated 24 h records. The estimates of FoPLs effects on food purchases were applied to the observational data in order to assess the nutrient content and food composition of a diet following a FoPL implementation (Fig. 1). A detailed presentation of the five FoPLs and the methods used is available as Additional file 1.

**Fig. 2:**
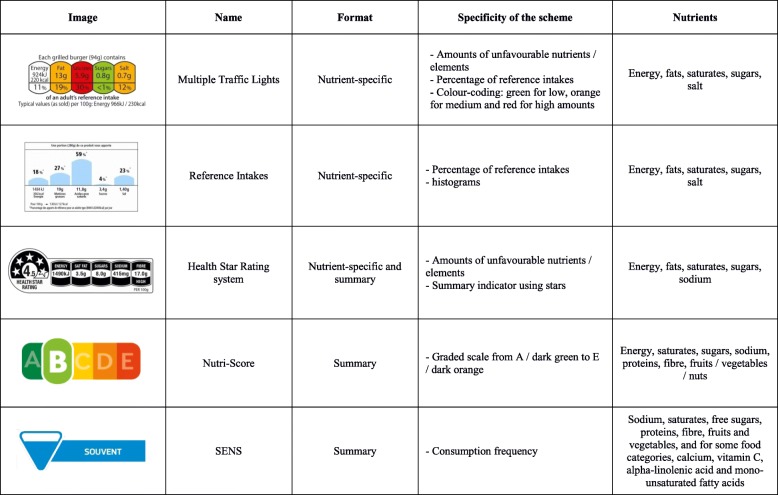
Front-of-pack nutrition labels tested

The frame-field experiment has been described in detail elsewhere [15, 16]. Briefly, the experimental study was used to determine relative differences in nutrient content and composition of food purchases between a reference situation with no FoPL and various labelling situations with one of the following five FoPLs affixed on food products: MTL, HSR, RIs, Nutri-Score, or SENS (Fig. 2). The study involved 691 adults recruited from the general population of the Grenoble metropolitan area, located in south-eastern France. Recruitment was performed in groups (sessions) of 20 participants. Participants were in charge of grocery shopping for their household and regular supermarket customers. The sample was stratified by household income, one third in each of the following categories: < 2000€/month, 2000–3000€/month, and > 3000€/month. Individual characteristics of this sample are summarised in Additional file 1: Table S1. Participants were asked to simulate their food purchases according to their usual shopping habits, without any information on the amount to spend, with the optional task of shopping for 2 days for their household. They were first asked to shop using a benchmark paper catalogue, including 290 food products from 39 food categories, without any FoPL on any of the food products. For each product, the following information was provided: a colour photograph, price, weight (g) or volume (L), price per kilogram or per litre, and a bar code. Using a bar-code reader, participants could display on their screen the product in a custom online e-shopping environment and access the list of ingredients and a nutritional facts table of the given food product. Then, participants were randomised to one of the six groups, with the standard practices of studies in the field [17]. The randomization unit was the session in which participants were recruited, using a drawing without replacement from an urn containing #treatments * #session, in this case 6 treatments * 7 sessions = 42 options. Hence, they were asked to shop a second time, using the same paper catalogue but this time one of the five FoPLs was displayed on each food product – except for fresh fruits and vegetables, fresh packaged meat, and eggs, according to European regulation – or a control catalogue identical to the one presented in the previous task, depending on the randomization arm. Before starting the experiment, participants were informed that they would actually need to purchase some of the items in one of their shopping carts, in order to display representative purchasing behaviours. Thus, at the end of the experiment, one of the two shopping carts of each participant was randomly selected and some of the products were actually purchased, depending on availability. Relative differences in nutrient content and composition of the food purchases (in terms of energy (kcal), fats (g), SFA (g), total sugars (g), fibre (g), salt (g), fruit (g), vegetable (g) were computed as the percentage change between the situation with a FoPL and the reference situation. Distribution of the effects of FoPLs on overall nutritional quality of household purchases was also investigated. First, mean relative differences in nutrient content and compositions of food purchases were calculated overall. Then, two variants were modelled, using the change in overall nutritional quality assessed by the modified Food Standard Agency-Nutrient Profiling System (FSAm-NPS) as quartiles [18]. Mean relative differences were calculated among participants in the first quartile of FSAm-NPS change, representing those having modified their purchases towards healthier choices (variant 1 – best case), and also among participants in the fourth quartile of FSAm-NPS change, representing those having modified their purchases towards unhealthier choices (variant 2 – worst case). Finally, to investigate the sex-specific effects (regarding sex of the person responsible for household purchases), relative differences among male and female shoppers only were assessed.

Observational data from participants in the NutriNet-Santé cohort (*N* = 81 421 participants, Additional file 1: Table S2) [19], were used to assess dietary intakes of the French population, from repeated 24 h dietary records of dietary consumption (fruit and vegetable) and nutrient intakes (energy, fats, SFA, fibre, and salt), thus yielding a reference diet, without any FoPL (data as observed, baseline scenario). For this purpose, volunteers of the cohort were invited to self-declare in real-time on a dedicated website, all food and beverages consumed during all eating occasion of the recording days. Dietary records were randomly assigned over a two-week period, with two weekdays and one weekend day. The relative differences observed in nutrient content and composition of household food purchases between the reference situation and the labelled situation were transposed to the dietary intakes of the sample, in order to estimate a ‘labelled’ diet (counterfactual scenario, corresponding to the hypothetical diet modified after introduction of one of the five different FoPLs). For example, the Nutri-Score was associated with a decrease of 9.04% of calories, which translated into a labelled diet consisting of 1797.6 kcal, as opposed to a reference diet of 1976.3 kcal. Dietary consumption and nutrient intakes were computed by sex and five-year age groups.

Data from the reference and ‘labelled’ diets were introduced in a macro-simulation model, the Preventable Risk Integrated ModEl (PRIME) [12]. The PRIME model does not simulate transitions over time, but rather compares the number of nutrition-related deaths associated with the dietary intakes in the baseline and counterfactual situations. The elements introduced in the model include the age and sex distribution of the French population (data derived from the 2014 Census), age and sex distribution of deaths by cause in the said population and age and sex distribution of dietary intakes for the baseline and counterfactual situations (derived from the frame-field experiment and NutriNet-Santé observational study). First, the model simulates number of deaths in the case of the baseline distribution of dietary intakes (reference diet scenario), assessed using observed dietary data of the NutriNet-Santé cohort sample (weighted in order to provide an estimated diet similar to the general French population) and computed by sex and five-year age brackets. Next, the model estimates number of deaths, using this time the counterfactual distribution of dietary intakes (‘labelled diet’ scenario). Thus, the estimated number of deaths averted or delayed from chronic diseases owing to the transition from a reference to a labelled diet is calculated using the difference in number of deaths between the two distributions.

Parameters for the baseline distribution introduced in the PRIME model were: mean total energy intake (kcal/d), mean and standard deviation (SD) of fruit consumption (g/d), percentage of participants consuming less than one fruit portion daily, mean and SD of vegetable consumption (g/d), percentage of participants consuming less than one vegetable portion daily, mean and SD of fibre intake (g/d), mean and SD of salt intake (g/d), mean and SD of total fat, SFA, MUFA and PUFA intakes (% of total energy), and mean and SD of dietary cholesterol intake (mg/d). The counterfactual distribution was determined by applying the specific relative difference values from the framed-field experiment, to the corresponding baseline dietary consumption: energy, fruit, vegetable, fibre, salt, fat, and SFA. Associations between nutrient intakes from diet and chronic diseases were parametrised in the PRIME model using meta-analyses of epidemiological studies providing estimates of relative risks linking specific nutrient intakes and disease outcomes (e.g. coronary heart disease relative risk per 106 g increase in fruit intake). All relative risk estimates obtained from meta-analyses and used in the PRIME model are reported elsewhere [12]. To allow these estimates to vary according to the distribution reported in the accompanying literature, uncertainty analysis using Monte Carlo simulations was performed to estimate credible intervals, for which 5th, 25th, median, 75th and 95th percentiles were used to model distribution of the results.

Data on mortality from nutrition-related chronic diseases were obtained from the International Statistical Classification of Diseases and Related Health Problems provided by the Epidemiological Centre on Medical Causes of Death in 2014 [20], providing exhaustive data for causes of deaths in France, and stratified by sex and five-year age groups. The age and sex structure of the population for the same year was determined using data from the French National Institute of Statistics and Economic Studies in 2014 [21]. A few different situations were tested in the simulation model regarding the relative differences applied to dietary intakes: mean relative differences of shopping carts nutrient content and food composition calculated overall, and mean relative differences in variants 1 (best case) and 2 (worst case). Moreover, to investigate effects associated with sex of the person usually responsible for household purchases, which are generally imposed on the whole household, sensitivity analyses were performed in which mean relative differences were calculated specifically among male and among female shoppers (see Additional file 1 for more details).

## Results

For each nutrient and FoPL format, differences in nutritional content of the shopping carts in the reference and labelled situations are presented in Table 1. Overall, FoPLs were associated with a decrease in the amount of energy, fat, SFA, and salt, − except for the SENS label -, and an increase in fibre and vegetable - except for the MTL label. Nutri-Score, HSR, and RIs were associated with a higher content of fruit, whereas MTL and SENS were associated with a lower content of fruit. Results were not uniform across FoPLs and nutrient-dependent. For most nutrients, differences were not statistically significant between the Nutri-Score, MTL and HSR, while each of these labels often differed significantly from the control group. Marked variability in consumer response was found for each FoPL and also in the control situation. This variability corresponded to both the effect of a specific FoPL and an overall heterogeneity in consumer behaviour across purchasing situations given the substantial number of possible food choices. Such variability is also apparent in variants 1 and 2 of the study. Compared with the control situation, all FoPLs – except HSR – were associated with a reduction in the heterogeneity of responses.

**Table 1:** Mean differences in nutritional content of the shopping carts between the reference situation (no label) and the labelled situation (one of five FoPL or no label)

	MTL	HSR	RIs	Nutri-Score	SENS	Control
Mean differences (%)						
cEnergy	−6.36^a,c^	−4.77^a,b^	−2.99^b,c^	−9.04^a^	−2.39^b,c^	−0.7^b^
Fats	−17.59^b^	− 14.63^b,c^	−9.1^c^	−21.38^b^	− 9.59^c^	0.75^a^
Saturated fatty acids	−24.01^b,c^	−19.83^c,d^	− 14.17^d^	−29.89^b^	−11.1^d^	1.63^a^
Salt	−5.39^b,c^	−7.1^b^	−3.41^a,b^	−4.1^b,c^	1.29^a,c^	3.29^a^
Fibre	0.86^b,c^	10.77^a^	2.41^b,d^	7.21^a,c,d^	9.71^a^	−0.99^b^
Fruit	−4.08^b^	6.19^a,b^	10.14^a,c^	12.36^a^	−0.01^b,c^	3.67^a,b^
Vegetable	−0.87^a^	2.81^a^	4.89^a^	5.38^a^	1.7^a^	3.54^a^
Variant 1 (best case): mean differences among participants in the first quartile of difference in FSAm-NPS (%)						
Energy	−9.09^a,b^	−6.4^a,b^	−5.31^a,b^	−13.42^a^	−6.99^a,b^	−2.32^b^
Fats	− 22.7^a^	− 23.66^a^	− 20.37^a^	− 27,00^a^	−24.31^a^	− 6.77^b^
Saturated fatty acids	− 31.68^b,c^	− 30.69^b,c^	− 28.4^b,c^	− 41.19^b^	− 21.83^a,c^	−7.97^a^
Salt	− 6.86^a,b^	− 11.54^b^	−10.19^a,b^	−6.88^a,b^	− 3.45^a,b^	2.02^a^
Fibre	3.36^b^	17.36^a^	4.23^a,b^	11.21^a,b^	9.16^a,b^	2.36^b^
Fruit	−5.87^b^	22.1^a^	4.27^a,b^	11.93^a,b^	−1.52^b^	9.99^a,b^
Vegetable	1.12^a^	7.00^a^	15.19^a^	10.98^a^	3.55^a^	10.18^a^
Variant 2(worst case): mean differences among participants in the fourth quartile of difference in FSAm-NPS (%)						
Energy	−2.82^a,b^	−1.04^a,b^	0.64^a,b^	−7.16^a^	2.24^a,b^	7.19^b^
Fats	−13.12^b^	−0.33^b^	2.58^a,b^	−10.75^b^	2.79^b^	18.77^a^
Saturated fatty acids	−14.78^a^	−2.1^a^	−4.75^a^	−12.98^a^	2.98^a^	26.15^b^
Salt	−2.45^b^	−1.02^b^	5.22^a,b^	−2.32^b^	5.34^a,b^	14.73^a^
Fibre	−5.49^a^	−1.11^a^	0.64^a^	−4.83^a^	6.44^a^	−3.78^a^
Fruit	−15.25^a^	−9.85^a^	5.3^a^	0.14^a^	− 3.34^a^	1.30^a^
Vegetable	−6.05^a^	−0.7^a^	−5.41^a^	−3.14^a^	−6.24^a^	3.80^a^

*MTL* Multiple Traffic Lights, *HSR* Health Star Rating, *RIs* Reference Intakes, *FSAm-NPS* Food Standards Agency modified Nutrient Profiling System; SENS: *Système d’Etiquetage Nutritionnel Simplifié*

^a, b, c, d^Means values with the same letter are not significantly different (Tukey’s multiple comparisons tests with a significance threshold of *p* < 0.05)

Results are expressed as percentages

**Fig. 3:**
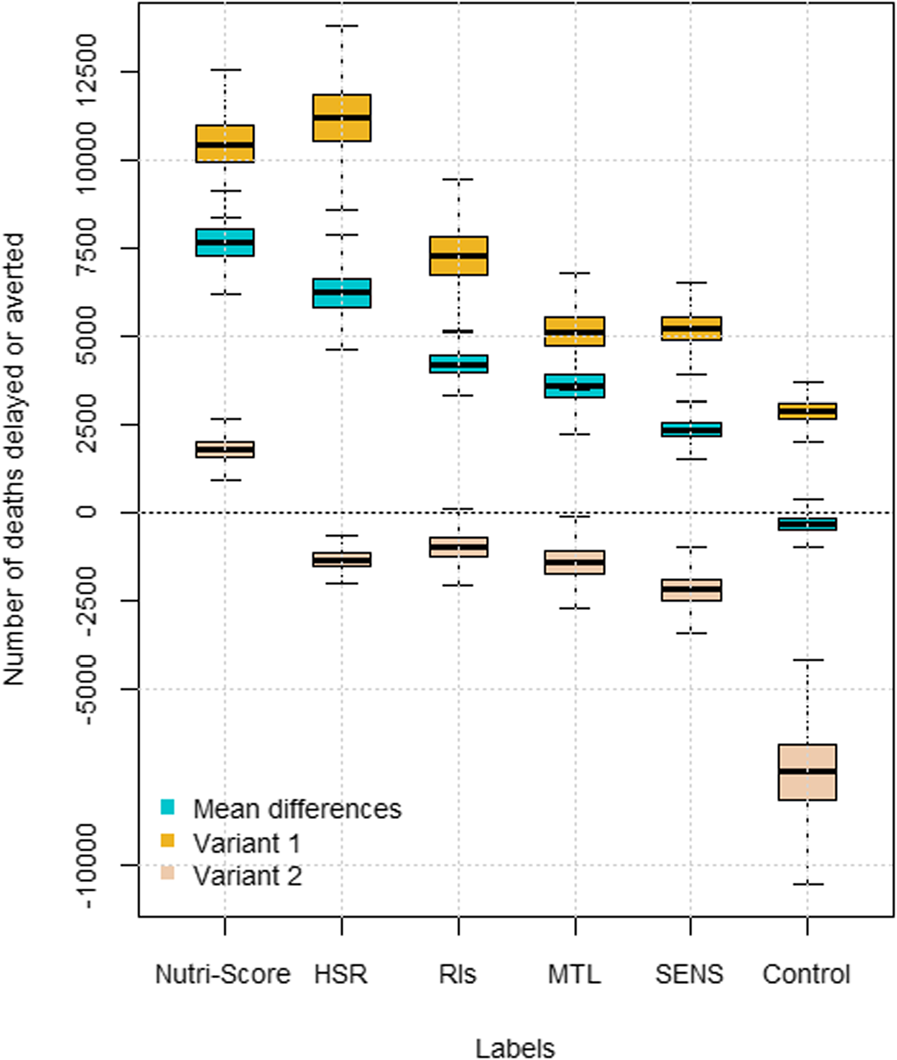
Number of deaths avoided through the use of FOP labels (and control situation). MTL: Multiple Traffic Lights; HSR: Health Star Rating; RIs: Reference Intakes; SENS: *Système d’Etiquetage Nutritionnel Simplifié*. Blue: Mean scenario overall; Beige: variant 1 (best case - mean differences in the first quartile of difference in FSAm-NPS); Yellow: variant 2 (worst case - mean differences in the fourth quartile of difference in FSAm-NPS)

Using the mean differences as the counterfactual scenario in the PRIME model, modifications in dietary intakes through FoPLs resulted in 2365 (95% credible interval: 1761 to 2975) for SENS and up to 7680 (6636 to 8732) for Nutri-Score averted or delayed deaths from chronic diseases (Fig. 3, Additional file 1: Table S3). Results for Nutri-Score corresponded to approximately 3.4% of all deaths from diet-related chronic diseases that were averted or delayed, followed by HSR (2.8%; 6265 (5115 to 7409) deaths), RIs (1.9%; 4223 (3569 to 4886) deaths), MTL (1.6%; 3583 (2657 to 4532) deaths), and SENS (1.1% of deaths averted). In variant 1, similar trends were observed, with higher numbers of deaths averted or delayed; however, in this variant, the HSR system slightly outperformed the Nutri-Score (5.0%; 11231 (9350 to 13104) deaths vs. 4.6%; 10488 (8976 to 11967) deaths). Relative ranking of the other labels remained largely unchanged. In variant 2, the Nutri-Score was the only FoPL shown to have a substantial impact regarding averting or delaying deaths from chronic diseases (0.8%; 1808 (1143 to 2446) deaths), while the other labels led to an increase in the number of deaths compared with the reference situation.

Among the various chronic diseases, mortality from cardiovascular diseases was the most impacted by modifications in diet induced by FoPLs (Table 2). More specifically, the main chronic diseases with a reduced mortality through the use of FoPLs were coronary heart disease, stroke, heart failure, hypertensive disease, and lung and colorectal cancers.

**Table 2:** Potential reduction in mortality by the use of FoPLs, by principal cause and by label

	MTL	HSR	RIs	Nutri-Score	SENS	Control
Mean differences (number of deaths)						
Total	3583 (2657 to 4532)	6265 (5115 to 7409)	4223 (3569 to 4886)	7680 (6636 to 8732)	2365 (1761 to 2975)	− 307 (− 826 to 168)
Cardiovascular disease	3151 (2250 to 4090)	5246 (4162 to 6391)	3517 (2910 to 4141)	6189 (5220 to 7197)	1823 (1269 to 2386)	− 458 (− 968 to 16)
Cancer	103 (−11 to 221)	770 (486 to 1024)	548 (339 to 743)	1030 (713 to 1332)	416 (198 to 615)	113 (38 to 184)
Variant 1 (best case): mean differences among participants in the first quartile of difference in FSAm-NPS (number of deaths)						
Total	5158 (3940 to 6400)	11231 (9350 to 13104)	7336 (5814 to 8909)	10488 (8976 to 11967)	5226 (4287 to 6186)	2880 (2247 to 3472)
Cardiovascular disease	4482 (3291 to 5695)	9317 (7572 to 11157)	6561 (5067 to 8134)	8525 (7095 to 9955)	4355 (3452 to 5274)	2241 (1626 to 2807)
Cancer	213 (29 to 397)	1583 (981 to 2128)	498 (356 to 629)	1298 (922 to 1645)	511 (285 to 716)	516 (297 to 719)
Variant 2 (worst case): mean differences among participants in the fourth quartile of difference in FSAm-NPS (number of deaths)						
Total	− 1414 (− 2404 to − 450)	− 1342 (− 1820 to − 859)	− 983 (− 1799 to − 224)	1808 (1143 to 2446)	− 2186 (− 3131 to − 1302)	− 7389 (− 9755 to − 5237)
Cardiovascular disease	− 832 (− 1713 to 58)	−995 (− 1403 to − 578)	− 1130 (− 1958 to − 383)	1341 (706 to 1970)	− 2062 (− 2978 to − 1183)	− 6602 (− 8976 to − 4467)
Cancer	−732 (− 1129 to − 311)	−403 (− 642 to − 160)	181 (72 to 286)	98 (− 24 to 229)	− 1 (− 170 to 161)	− 382 (− 491 to − 271)

*MTL* Multiple Traffic Lights, *HSR* Health Star Rating, *RIs* Reference Intakes, *FSAm-NPS* Food Standards Agency modified Nutrient Profiling System; SENS: *Système d’Etiquetage Nutritionnel Simplifié*

Results are expressed as number of deaths

Results were robust, as seen when taking into account the sex of the main grocery shopper in the household. Using data from male shoppers only, we observed a relative increase in the number of delayed or averted deaths from chronic diseases for MTL, HSR, and RIs, and a decrease for Nutri-Score and SENS, compared to the overall scenario (Additional file 1: Table S4). Nonetheless, HSR (3.2%; 7321 (5749 to 8875) deaths) and Nutri-Score (3.2%; 7280 (6298 to 8210) deaths) remained the two FoPLs with the highest impact on deaths averted. Using data from female shoppers only we observed an increase in the number of deaths delayed or averted for Nutri-Score and SENS, and a decrease for MTL, HSR, and RIs compared with the overall scenario. Again, the Nutri-Score performed best (3.4%; 7765 (6657 to 8837) deaths), followed by the HSR (2.6%; 5965 (4870 to 7077) deaths).

## Discussion

Results of the present study were based on the effects of FoPLs on the nutritional quality of food purchases, estimated using an experimental study. All FoPLs tested improved the nutritional quality of the shopping carts, with a decrease in the amount of energy, fats, and SFA, and an increase in fibre; most labels led to a decrease in the amount of salt (except for SENS), an increase in fruit (except for MTL and SENS), and vegetable (except for MTL). Results of the FoPLs effects on the nutritional quality of food purchases were consistent with those of other studies, which have found positive effects of FoPLs such as the Nutri-Score and MTL on nutritional quality of purchases [22–25]. However, the FoPLs effects observed in the present study appeared of higher magnitude compared to other studies. Using the PRIME model, we observed that FoPLs may lead to up to 3.4% of deaths averted or delayed by chronic diseases, on average. However, results were dependent on label format, with the highest estimates obtained for Nutri-Score and HSR, which are both summary graded systems.

Overall, the effects observed in the modification of food purchases and diets were reflected in the total number of deaths avoided or delayed, with stronger improvements in diets (i.e. larger differences) translating into a larger number of deaths avoided, and an overall neutral effect in the control situation. However, the simulated impact on health that was observed in the case of MTL remained limited. Even though improvement in dietary intakes appeared higher than that observed with other FoPL designs regarding some nutrients (e.g. energy, fats, SFA, and salt), their overall performance did not entirely align. This may be related to their underlying nutrient profiling system and the information provided to consumers. More specifically, MTL only highlights unfavourable nutrients (e.g. fat, SFA, sugars, salt). Therefore, even though MTL can lead to a higher decrease in the consumption of these nutrients, they can also lead to weak increases in intake of favourable nutrients, such as fibre, fruit and vegetable. However, in this study, it appeared that an increase in fruit and vegetable consumption had a particularly strong impact on mortality from NCDs compared with a modification in unfavourable nutrient consumption, as has been observed in other studies [26–29]. Such findings highlight the importance of taking into account favourable elements, and in particular fruit and vegetable as key elements within the nutrient profiling system of a FoPL, as is the case with Nutri-Score and HSR.

Moreover, results from variants 1 and 2 emphasized the large variability in consumer response in two consecutive purchasing situations, in the context of a substantial number of food choices. However, except for the HSR system, FoPLs appeared to somewhat reduce that variability. Particularly, in the case of Nutri-Score, modification in dietary intakes in the labelled situation consistently led to a substantial number of deaths avoided (1808 (1143 to 2446) deaths). This finding might be explained by the graphical design of this FoPL, which is a summary graded indicator with colours with a high symbolic value (green – red), which has been demonstrated to be easier to read and understand compared with other formats [30–33]. Compared with Nutri-Score, the HSR system was associated with a higher variability in consumer response, and a larger difference in the number of deaths averted or delayed in variants 1 and 2. This specific result may partly be explained by the fact that the HSR format includes a higher number of categories of nutritional quality (from half a star to five stars in half-star increments which results in ten categories) compared with the A to E (five categories) for the Nutri-Score. The overall number of available categories featured on a FoPL might lead to a higher variability in consumer behaviour. Overall, these results suggest that some key elements of the Nutri-Score may explain its better performance compared with other formats. Such elements pertain to the inclusion of fruit and vegetable within its algorithm, the summary, graded graphical design, and inclusion of five categories (compared to three for MTL and ten for HSR) as a balanced number of categories from which to compare products for consumers. Some of these key features, such as the fruit and vegetable component of the algorithm and the summary and graded indicator, may also explain the satisfactory performance of the HSR.

To the best of our knowledge, no other study has investigated the effect of FoPL use on long-term health status. However, some studies have shown a positive effect of FoPLs on the nutritional quality of food purchases, leading to lower amounts of fats, SFA, sodium, and sugars, and higher amounts of fibre and protein, depending on the label format [5, 34–36]. Some label formats, such as those using colour-coding [5, 37–42] or warning symbols [42–44], may have an increased impact on product healthfulness identification and consumer food choices. Moreover, use of FoPLs has been suggested to be associated with nutrient intakes and the quality of diets [45, 46]. Finally, previous studies simulating the effects of the use of Nutri-Score or MTL in substitution scenarios have suggested that it would increase the nutritional quality of the diet, in particular for individuals with unhealthier diets [46, 47]. Observational studies using the underlying algorithm of the Nutri-Score as an indicator of the nutritional quality of individual diets have suggested that a higher nutritional quality of the foods consumed was associated with a lower incidence of nutrition-related chronic diseases (e.g., cancer, cardiovascular diseases, obesity, metabolic syndrome) [5]. Although these studies provided some indication of the potential impact of FoPLs – and of Nutri-Score in particular – on health outcomes, to date, no estimates of the actual impact of FoPLs on health using results from intervention trials are available.

Our study is the first to assess the direct impact of a public health measure such as FoPLs on mortality from chronic diseases. Some studies have investigated the impact of public health policies on mortality from NCDs using similar macro-simulation models. For example, a study conducted in the United Kingdom estimated that the achievement of the dietary recommendations could lead to approximatively 14% reduction in mortality from nutrition-related chronic diseases [29]. Similarly, a simulation study on the reduction of alcohol intake in the United Kingdom to 5 g/day resulted in a decrease of 3% mortality from partially alcohol-related chronic diseases [48], while another study simulated that the Danish saturated fat tax would decrease by 0.4% deaths from NCDs [27]. Compared to these simulations, investigating the impact of the adherence to nutritional recommendations, the implementation of a FoPL may represent an efficient public health strategy, with a substantial reduction in mortality from NCDs. Moreover, beside its immediate effects on consumer purchases, implementation of a FoPL might entice manufacturers to improve the nutritional quality of the food offer, through innovation and reformulation, which would further increase the ultimate impact of the FoPL [7, 8]. Furthermore, FoPLs appear to be a cost-effective strategy, as modelling studies have suggested that the adoption of a nutrition labelling would achieve both health gains and cost savings [13]. In particular, as the implementation of the FoPL relies on manufacturers rather than governments, the cost of adopting such a system would mainly rest on food companies.

One of the major strengths of the present study is its ability to fill knowledge gaps by providing for the first time an estimate of deaths number averted or delayed from chronic diseases linked to FoPL use. Furthermore, the study compared the impact of different FoPL formats, including nutrient-specific and summary labels. Furthermore, the study compares the impact of different FoPL formats, including nutrient-specific and summary labels. At the time of the frame-field experiment, the Nutri-Score was not yet implemented in the French market, excluding any potential bias related to familiarity. The Reference Intakes, which were already implemented by some manufacturers in French supermarkets, was the only FoPL with which participants might have had some familiarity, although a modified version of the scheme was used.

Some limitations of the study should be acknowledged. First, in the absence of data on the long-term effects of the five different FoPLs on dietary behaviour, we relied on estimates generated from an experimental study in controlled conditions. The experimental protocol may not have captured long-term changes in dietary behaviour. Likewise, the protocol did not account for evolution in the food offer through reformulation. However, to date, no study has provided estimates of long-term modifications in dietary behaviour related to the FoPLs tested. Even though a recent meta-analysis provided estimates of the overall impact of food labelling on purchases, it did not include studies on the Nutri-Score, nor did it provide estimates depending on the formats tested [25]. Such omissions may be related to the relatively recent introduction of some of the labels (the Nutri-Score was implemented in late 2017, the HSR in 2014) or to the format’s experimental nature (e.g. the SENS and modified RIs have not actually been implemented). The experimental study used here estimated the effects of these five different formats using a robust and standardised method across all FoPLs. Uncertainty in the estimated effects was handled using Monte Carlo simulations and variants to the mean modifications in food purchases. Another limitation is the use of food purchase data rather than consumption data to determine relative differences between the reference diet and the ‘labelled’ diet. However, some studies have suggested that purchases are a valid indicator of dietary patterns [49], thus any bias might be mitigated.

Some limitations related to the experimental methodology should be mentioned. First, the population sample included in the experiment was not representative of the French population. However, recruitment targeted a wide range of socio-demographic profiles. Thus, caution is needed regarding extrapolation of the results to the general French population. Nevertheless, the experimental study used here estimated the effects of these various formats using a robust and standardised method across all FoPLs. Moreover, the use of such data allowed consistency between the three parts of the study, all focusing on French participants. Complementary studies should be conducted in other countries as current findings may not be applicable in other nations. Next, in the frame-field experiment, purchases were performed at the household level, and we were not able to link the purchased products specifically to the consumer. The experimental study simulated the impact of FoPLs in a situation where all food products were labelled. Given that implementation of FoPL may be voluntary, the experimental design may have led to an overestimation of the effect of the labels on purchasing intentions and dietary behaviour. However, inclusion of variants to the mean purchasing behaviour allowed us to investigate variability of consumer responses regarding each FoPL, and thus provide estimates of the maximum and minimum impact that may be expected. Moreover, in the present study the effect of the FoPLs on mortality was investigated according to the sex of the shopper, while other individual characteristics (e.g. body mass index, interest in nutrition.) might have influenced the observed effect. However, the impact of the labels on food purchases according to other variables was unknown.

Some limitations related to the PRIME model use should be also mentioned. First, the parametrization of this macro-simulation model is limited by the present availability of robust meta-analyses estimating relative risks for mortality from specific chronic diseases. However, uncertainty was mitigated by the performance of Monte Carlo simulations, allowing the association parameters to vary stochastically according to distributions reported in the literature. It is also important to mention that the PRIME model estimated the impact of FoPLs use on mortality, and did not provide estimates of impact on morbidity from diet-related chronic diseases. However, both morbidity and mortality contribute to the high burden of poor diet quality on current health systems. Moreover, the PRIME model, similar to other non-communicable diseases scenario models, does not take into account interactions among behavioural risk factors for chronic diseases, mostly due to lack of empirical evidence. In addition, the PRIME model does not incorporate the effect of time lag between exposure and chronic diseases outcome, and exposure is considered constant over time as other macro-simulation models. Nevertheless, the three situations tested in the model (mean differences, variant 1 – best case, and variant 2 – worst case) allowed us to assess the impact of different labels’ effects magnitudes on mortality. Finally, it is important to note that application of the results of the present study regarding notably the superiority of the Nutri-Score would depend on a mandatory implementation of the label on food products (presently, it is used on a voluntary basis, as per European Union regulations). Nevertheless, more than 100 manufacturers already committed themselves to apply the Nutri-Score in French supermarkets, corresponding roughly more than 20% of market share.

## Conclusions

The present macro-simulation study suggests that the use of a FoPL may help prevent a large number of deaths, with label format-specific effects. The Nutri-Score, with its graded and summary format featuring semantic colours appears to be the most efficient FoPL in terms of decreasing mortality from diet-related NCDs (up to 3.4% on average), including in individuals with a low response to FoPLs. These results strengthen interest in the choice of Nutri-Score as an effective tool in public health, to improve nutritional status of populations and prevent chronic diseases.

## Additional file

Additional file 1: This supplemental material provides additional details on the methodologies of the present study, as well as supplemental figures and tables of results. (DOCX 484 kb)

## Data Availability

All data supporting the findings of this study are included in the present article or the supplemental material.

## Abbreviations

FoPL: Front-of-Pack Label
FSAm-NPS: modified Food Standard Agency-Nutrient Profiling System
HSR: Health Star Rating
MTL: Multiple Traffic Lights
NCD: Non-Communicable Disease
PRIME: Preventable Risk Integrated ModEl
RIs: Reference Intakes
SD: Standard Deviation
SENS: *Système d’Etiquetage Nutritionnel Simplifié*
SFA: Saturated Fatty Acids
UK: United Kingdom
