# Supplementary material for: Modelling the impact of different front-of-package nutrition labels on mortality from non-communicable chronic disease
Source: Int J Behav Nutr Phys Act. 2019 Jul 15;16:56. doi: 10.1186/s12966-019-0817-2 (PMC6631735; doi:10.1186/s12966-019-0817-2)
Supplement: Supplementary file 1 — This supplemental material provides additional details on the methodologies of the present study, as well as supplemental figures and tables of results. (DOCX 484 kb) [file 12966_2019_817_MOESM1_ESM.docx]

**Supplemental Material**

**Modelling the impact of different front-of-package nutrition labels on mortality from non-communicable chronic disease**

Table of Contents

[Section S1. Additional methodological details 2](#_Toc172715)

[Figure S1. Front-of-pack nutritional labels tested 8](#_Toc172716)

[Table S1. Socio-demographic characteristics of the experimental economics population study (N=691) 8](#_Toc172717)

[Table S2. Socio-demographic characteristics of the NutriNet-Santé sample (N=81 421) 9](#_Toc172718)

[Table S3. Number of deaths avoided through the use of FoPLs (and control situation) 9](#_Toc172719)

[Table S4. Number of deaths avoided through the use of FoPLs (and control situation) using differences by sex 10](#_Toc172720)

# Section S1. Additional methodological details

**Materials and methods**

**Data used to test the impact of FoPLs on food purchases**

In November 2016, a laboratory framed-field experiment was conducted in France to assess the impact of Front-of-Pack nutrition labels (FoPLs) on food purchases under laboratory conditions. Complete information about the study can be found elsewhere.(1,2) Briefly, the study aimed to investigate the nutritional quality of food purchases in a sample of consumers before and after the implementation of five different FoPLs on food products: Multiple Traffic Lights (MTL), Health Star Rating (HSR) system, a modified version of Reference Intakes (RIs), Nutri-Score and SENS (**Figure S1**).

The MTL, introduced by the United Kingdom Food Standards Agency (FSA), and the RIs are two nutrient-specific labels providing numeric information on the nutrient content for a portion of a product, regarding energy, total fat, saturated fatty acids, sugars, and sodium, and their contribution within an adult’s diet (calculated taking into account the reference intakes set by the European Union regulation on food labelling(3)). The MTL also adds colours (green, amber, red), depending on the amount of the various nutrients in the product (low, moderate, high).(4) The attribution of colours is based on the content of each nutrient per 100g or 100ml of the product. For the RIs, the percentage of reference intakes for a portion is represented in the form of histograms.(5) The RIs were developed by the French agro-industry trade association.

The Nutri-Score, the Health Star Rating system and the SENS are summary graded systems, providing a single indicator of the nutritional quality of the product. The Nutri-Score, composed by a graded five-colours scale associated to letters (from dark green (A) for highest nutritional quality to red (E) for lowest nutritional quality)(6) was selected by French health authorities in 2017 to be applied on foods in the French market.(7) The HSR system, represented by a rate from half a star to five stars, was implemented in Australia and New Zealand in 2014.(8) Finally, the SENS is a four-category summary label indicating the recommended consumption frequency of a given food item (green-labelled food could be eaten “very often”, blue “often”, orange “regularly in small quantities” and purple “occasionally or in small quantities”)(9) and was developed by the French retailers trade association.

To be included in the study, participants had to be in charge of the grocery shopping for their household and had to usually purchase food products in supermarkets. Furthermore, recruitment was stratified according to the level of income, in order to ensure representative consumer behaviours. Thus, three groups of income level were constituted: <2000€/month, 2000-3000€/month, and >3000€/month. Socio-demographic data were collected at the end of the shopping session. Individual characteristics of the population of the study are presented in **Table S1**.

An experimental supermarket was developed on a computer platform and each participant had to shop a two days’ worth of food for their household. The experimental supermarket included 290 food products, divided into 39 categories, including raw foods, and was presented to the participants in a paper colour catalogue. Participants were equipped with barcode readers and could use the catalogue as a basis for later on-screen shopping. Each food product was available in real supermarkets at the time of the study, and the prices displayed in the catalogues reflected those in a Grenoble area supermarket in the previous weeks. For each food product, the nutritional composition and the ingredient list were previously determined and collected through supermarket surveys, and available on screen. To ensure that purchases reflected real purchasing behaviours, about a quarter of the food products bought by the participants were actually sold to each participant at the end of the experiment.

Each participant was invited to constitute a first shopping cart using a catalogue displaying no FoPL. This was considered as the reference cart. Then, participants were randomly assigned to one of five intervention groups (with one of the five FoPLs above applied on food products) or in a control group with no FoPL on products, and were invited to create a new shopping cart. At the end of the experiment, one of the two carts was randomly selected to be binding, and participants had to purchase the product they had selected, subject to availability.

The impact of FoPLs on the overall nutritional quality of food purchases was assessed using the mean FSAm-NPS (Food Standard Agency modified - nutrient profiling system) of the items in the shopping cart.(6,10) The FSAm-NPS is calculated for 100g of product and allocates positive points, zero to ten points for each nutrient which intake has to be limited (i.e. energy (kJ), saturated fatty acids (g), sugars (g), and sodium (mg)), and negative points, zero to five points to each favourable nutrient (i.e. proteins (g), fibre (g) and the content of fruits, vegetables and nuts (%)). FSAm-NPS of the shopping cart was calculated taking into account the weight of the product in the cart. A lower FSAm-NPS reflects a higher nutritional quality of the shopping cart. The nutritional content of the shopping carts was calculated in each FoPL group, in the reference situation and in the labelled situation for each participant. To investigate the effects of FoPLs on diets, the effects observed on the shopping carts were transposed to diets, with the assumption that the differences observed before and after the implementation of FoPLs on nutritional quality of food purchases were directly passed on dietary consumptions.

To that effect, a two-step procedure was used. First, the nutrient content of the foods purchased were transposed from foods as sold to foods as consumed, using standard dietary procedures (e.g. removal of edible part, rehydration rate for dried products). Second, for each participant in the experimental study, the relative differences between the reference and the labelled situation in each arm of the study were computed for each of the nutrients (energy, fats, saturated fatty acids (SFA), salt, fibre, fruits, and vegetables) and the FSAm-NPS, using composition data of foods as consumed. To avoid outlier behaviour, participants whose relative difference between the reference and the labelled situation was lower than the 5^th^ percentile or higher than the 95^th^ percentile, were excluded from the computation of the difference for the corresponding nutrient. Nutrient content and composition of the shopping carts were estimated per person by dividing the total amount of purchases by the number of household members, weighted by consumption unit in the household.(11) We also assumed that all purchased food products were consumed by all the members in the household. This procedure defined the relative differences in individual nutrient intakes between a reference situation and a labelled situation, which were directly applied to diets.

**Data used for application to dietary consumptions**

**Population study**

Assuming that effects of FoPLs on food purchases can be reflected on dietary consumptions, relative differences calculated between the two shopping carts were applied on dietary consumptions collected in the NutriNet-Santé cohort, in order to set a ‘reference’ diet (dietary intakes as observed, without any FoPL) and a ‘labelled’ diet for each FoPL. Complete information about the NutriNet-Santé study has been published elsewhere.(12) Briefly, the NutriNet-Santé study is a French ongoing web-based cohort, involving volunteer participants over 18 years old, recruited since 2009 by multimedia campaigns. At inclusion and during their follow-up, participants are invited to fill online questionnaires, assessing dietary intakes, anthropometric characteristics, lifestyle, socioeconomic conditions, physical activity, and health status.

For the present study, socio-demographic and lifestyle data collected were sex, age, self-reported height and weight, body mass index (BMI), smoking status, and physical activity (through the use of the international validated IPAQ questionnaire).(13) Participants of the NutriNet-Santé cohort who had filled three dietary records at inclusion and had no missing data for the socio-demographic and lifestyle variables were included. Data were weighted so that the sample was representative of the French population socio-demographic distribution using the SAS CALMAR macro developed by the French National Institute of Statistics (INSEE). Weighting was calculated separately by sex using the national Census data of 2009 on age, educational level, occupational category, area of residence, presence of children in the household, and marital status.(14) Characteristics of the NutriNet-Santé sample are described in **Table S2**.

**Dietary consumption data**

Dietary intakes were assessed at inclusion in the participants of the cohort between 2009 and 2016, using three non-consecutive validated web-based 24h-dietary records, randomly assigned over a two-week period, including two weekdays and one weekend day.(15–17) All foods and beverages consumed during a 24h-period were declared on a specific interface and portion sizes were estimated using validated photographs.(18) Mean daily energy, nutrient and alcohol intakes were calculated using a published French food composition table, including more than 3 300 items.(19) Composite dishes were decomposed using French recipes validated by food and nutrition professionals. Dietary underreporting was identified through the method proposed by Black, and under-energy reporters were excluded.(20)

The dietary intakes as observed in the NutriNet-Santé study were used as the ‘reference’ diets. Then, the relative differences defined in the experimental study were directly applied to the observed dietary effects, to define ‘labelled’ diets, which were used as counterfactual scenarios in the PRIME model.

**PRIME model and estimation of the effect of FoPLs on mortality from NCDs**

The Preventable Risk Integrated ModEl (PRIME) is a macro-simulation model which was used to assess the impact of changing the distribution of nutrient consumptions on mortality from chronic diseases. The PRIME model takes into account dietary consumptions (including alcohol consumption) and nutrient intakes, physical activity, height, BMI, and smoking status.

The observed food consumption (fruit and vegetables) and dietary intakes (energy, fibre, salt, fats, SFA, monounsaturated fatty acids (MUFA), polyunsaturated fatty acids (PUFA), and cholesterol) in the NutriNet-Santé were used to determine the reference (baseline) and counterfactual diets introduced in the PRIME model.

**Mortality and socio-demographic data**

The list of chronic diseases related to diet and data of mortality from the selected chronic pathologies were obtained from the International Statistical Classification of Diseases and Related Health Problems provided by the Epidemiological Centre on Medical Causes of Death in 2014.(21) Chronic diseases defined as health outcomes in the PRIME model included cancers (ICD-10: C00-16, 18-20, C22-25, C34, C50, C53, C54.1, C64, C67), metabolic diseases (ICD-10: E11, E14), diseases of the circulatory system (ICD-10: I05-15, I20-26, I50, I60-69, I71), diseases of the respiratory system (ICD-10: J40-44), diseases of the digestive system (ICD-10: K70, K74), and diseases of the genitourinary system (ICD-10: N18). Data of mortality were stratified by sex and five-year age groups.

The age and sex structure of the population for the same year was determined using data from the National Institute of Statistics and Economic Studies in 2014.(22)

**Baseline and counterfactual scenario of dietary distribution**

The baseline distribution of the different nutritional intakes was assessed using dietary data of the NutriNet-Santé cohort sample and computed by sex and five-year age brackets. Parameters for the baseline distribution were: mean total energy intake (kcal/d), mean and standard deviation (SD) of fruit consumption (g/d), percentage of participants consuming less than one fruits portion daily, mean and SD vegetables consumption (g/d), the percentage of participants consuming less than one vegetables portion daily, mean and SD fibre intake (g/d), mean and SD salt intake (g/d), mean and SD total fat, SFA, MUFA and PUFA intakes (% of total energy), and mean and SD dietary cholesterol intake (mg/d).

The counterfactual distribution was determined by applying the specific relative differences from the framed-field experiment, to the corresponding baseline dietary consumptions: energy, fruits, vegetables, fibre, salt, fats, and SFA. For these nutrients, the assumption was to apply the relative differences in purchases observed in the experimental study between the reference situation and the labelled situation to the baseline dietary consumptions, in order to assess a ‘labelled’ diet. For fats, we assumed that the modifications observed on SFA intakes did not impact PUFA and MUFA intakes but only the relative distribution of the different fatty acids in the diet.

For the counterfactual scenario, we first applied the mean differences calculated in each FoPL group using the framed-field experiment data. Secondly, in order to investigate the variability in the responses to FoPLs, two variants based on the first and fourth quartiles of the difference in overall nutritional quality of purchases – using the FSAm-NPS of the shopping cart – between the reference and the labelled situation, were tested. In variant 1 (best case), mean differences calculated among participants from the first quartile were applied. These individuals are the ones who decreased the most the FSAm-NPS of their shopping carts, e.g. had the highest difference in nutritional quality (towards healthier purchases) between the reference and the intervention situations. In variant 2 (worst case), mean differences calculated among participants from the fourth quartile were used, corresponding to individuals who had the lowest (or negative) difference in nutritional quality towards unhealthier purchases between the reference and the intervention situations.

Finally, sensitivity analyses were carried out by testing two more variants for each FoPL: (1) mean differences were calculated in each FoPL group from the framed-field experiment among male shoppers only and then applied to the dietary consumptions of the NutriNet-Santé cohort participants, and (2) mean difference were calculated among female shoppers only and applied to the dietary consumptions.

**Parameterization of the association between dietary consumptions and chronic diseases**

Using relative risks of different levels of nutrient intakes on chronic diseases and the baseline distribution, the PRIME model estimates death rates associated to chronic diseases in a given situation. Then the model simulates the number of deaths averted or delayed from chronic pathologies, resulting from the difference of the averted deaths number between the baseline and counterfactual scenarios.

A full description of the PRIME model, including all the parameters used in the model, has been described in depth elsewhere.(23)

**Uncertainty analyses**

Each of the estimates of relative risks used in the model was allowed to vary according to the distribution reported in the accompanying literature. Thus, a Monte Carlo simulation was performed to estimate 95% credible intervals around results. Ten thousands iterations were run to produce the 2.5^th^, 25^th^, 75^th^, and 97.5^th^ percentiles of results.


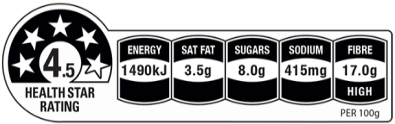

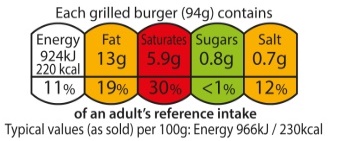

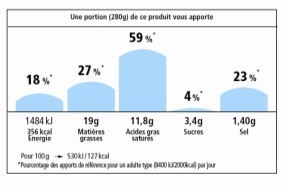


Multiple Traffic Lights

Health Star Rating system

Reference Intakes


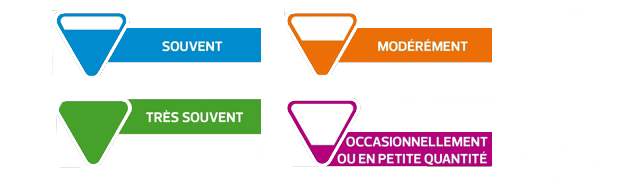


SENS


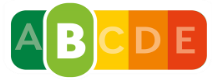


Nutri-Score / Santé Publique France 2017

# Figure S1. Front-of-pack nutritional labels tested

# Table S1. Socio-demographic characteristics of the experimental economics population study (N=691)

|  | **All** | **MTL** | **HSR** | **RIs** | **Nutri-Score** | **SENS** | **Control** |
| --- | --- | --- | --- | --- | --- | --- | --- |
|  | **N (%)** | **N (%)** | **N (%)** | **N (%)** | **N (%)** | **N (%)** | **N (%)** |
| **Total** | 691 | 115 (16.6) | 119 (17.2) | 109 (15.8) | 115 (16.6) | 111 (16.1) | 122 (17.7) |
| **Sex** |  |  |  |  |  |  |  |
| Men | 140 (20.3) | 22 (19.1) | 24 (20.2) | 23 (21.1) | 26 (22.6) | 18 (16.2) | 27 (22.1) |
| Women | 551 (79.7) | 93 (80.9) | 95 (79.8) | 86 (78.9) | 89 (77.4) | 93 (83.8) | 95 (77.9) |
| **Age** |  |  |  |  |  |  |  |
| 18 - 24 | 12 (1,7) | 1 (0,9) | 5 (4,2) | 2 (1,8) | 0 | 2 (1,8) | 2 (1,6) |
| 25 - 39 | 377 (54,6) | 61 (53,0) | 62 (52,1) | 57 (52,3) | 68 (59,1) | 55 (49,6) | 74 (60,7) |
| 40 - 54 | 255 (36,9) | 39 (33,9) | 43 (36,1) | 45 (41,3) | 41 (36,7) | 46 (41,4) | 41 (33,6) |
| 55 - 64 | 46 (6,7) | 13 (11,3) | 9 (7,6) | 5 (4,6) | 6 (5,2) | 8 (7,2) | 5 (4,1) |
| ≥ 65 | 1 (0,1) | 1 (0,9) | 0 | 0 | 0 | 0 | 0 |
| **Income level** |  |  |  |  |  |  |  |
| <2000 | 268 (38.8) | 46 (40.0) | 50 (42.0) | 40 (36.7) | 43 (37.4) | 40 (36.0) | 49 (40.2) |
| 2000-3000 | 212 (30.7) | 35 (30.4) | 38 (31.9) | 35 (32.1) | 36 (31.3) | 33 (29.7) | 35 (28.7) |
| >3000 | 211 (30.5) | 34 (29.6) | 31 (26.1) | 34 (31.2) | 36 (31.3) | 38 (34.2) | 38 (31.2) |
| **Educational level** |  |  |  |  |  |  |  |
| Primary | 178 (25.8) | 29 (25.2) | 25 (21.0) | 33 (30.3) | 26 (22.6) | 28 (25.2) | 37 (30,3) |
| Seconday | 94 (13.6) | 18 (16.7) | 19 (16.0) | 16 (14.7) | 20 (17.4) | 9 (8.1) | 12 (9.8) |
| University | 401 (58.0) | 65 (56.5) | 71 (59.6) | 57 (52.3) | 69 (60.0) | 69 (62.2) | 70 (57.4) |
| Other | 18 (2.6) | 3 (2.6) | 4 (3.4) | 3 (2.6) | 0 | 5 (4.5) | 3 (2.5) |
| **Profession** |  |  |  |  |  |  |  |
| Blue-collar, manual workers | 34 (4.9) | 0 | 4 (3.4) | 9 (8.3) | 3 (2.6) | 10 (9.0) | 8 (6.6) |
| Intermediate profession / office staff | 517 (74.8) | 92 (80.0) | 97 (81.5) | 75 (68.8) | 90 (78.3) | 83 (74.8) | 80 (65.6) |
| Managerial staff | 98 (14.2) | 13 (11.3) | 10 (8.4) | 22 (20.2) | 16 (13.9) | 14 (12.6) | 23 (18.8) |
| Without professional activity | 42 (6.1) | 10 (8.7) | 8 (6.7) | 3 (2.8) | 6 (5.2) | 4 (3.6) | 11 (9.0) |
| **Household** |  |  |  |  |  |  |  |
| With children | 578 (83.7) | 84 (73.0) | 103 (86.6) | 94 (86.2) | 97 (84.4) | 94 (84.7) | 106 (86.9) |
| Without children | 113 (16.3) | 31 (27.0) | 16 (13.4) | 15 (13.8) | 18 (15.6) | 17 (15.3) | 16 (13.1) |

MTL: Multiple Traffic Lights; HSR: Health Star Rating; RIs: Reference Intakes.

# Table S2. Socio-demographic characteristics of the NutriNet-Santé sample (N=81 421)

|  | **%** |
| --- | --- |
| **Sex** |  |
| Men | 47.7 |
| Women | 52.3 |
| **Age (years)** |  |
| 18 - 24 | 10.9 |
| 25 - 39 | 24.9 |
| 40 - 54 | 26.9 |
| 55 - 64 | 17.0 |
| ≥ 65 | 20.3 |
| **Educational level** |  |
| Primary | 55.4 |
| Secondary | 16.6 |
| University | 28.0 |
| **Occupational category** |  |
| Blue-collar, manual workers | 10.0 |
| Intermediate profession/office staff | 33.9 |
| Self-employed, farmer | 4.4 |
| Managerial staff | 10.2 |
| Without professional activity | 9.4 |
| Student | 5.0 |
| Retired | 27.1 |
| **Marital status** |  |
| Married | 52.0 |
| Partnership | 12.4 |
| Divorced | 8.2 |
| Widowed | 6.1 |
| Single | 21.3 |

# Table S3. Number of deaths avoided through the use of FoPLs (and control situation)

|  | **MTL** | **HSR** | **RIs** | **Nutri-Score** | **SENS** | **Control** |
| --- | --- | --- | --- | --- | --- | --- |
| **Mean differences** | | | | | |  |
| **Total** | 3583 (2657 to 4532) | 6265 (5115 to 7409) | 4223 (3569 to 4886) | 7680 (6636 to 8732) | 2365 (1761 to 2975) | -307 (-826 to 168) |
| **Under 75** | 1235 (997 to 1477) | 1997 (1697 to 2299) | 1405 (1195 to 1607) | 2495 (2175 to 2797) | 892 (716 to 1068) | -73 (-210 to 55) |
| **Male under 75** | 978 (793 to 1167) | 1499 (1266 to 1732) | 1049 (894 to 1200) | 1875 (1636 to 2099) | 670 (531 to 809) | -73 (-178 to 26) |
| **Female under 75** | 257 (201 to 315) | 498 (418 to 577) | 356 (296 to 410) | 620 (532 to 706) | 222 (171 to 272) | 0 (-33 to 31) |
| **Variant 1 : mean differences in the first quartile of difference in**  **FSAm-NPS** | | | | | |  |
| **Total** | 5158 (3940 to 6400) | 11231 (9350 to 13104) | 7336 (5814 to 8909) | 10488 (8976 to 11967) | 5226 (4287 to 6186) | 2880 (2247 to 3472) |
| **Under 75** | 1703 (1392 to 2022) | 3603 (3041 to 4126) | 2392 (2009 to 2775) | 3285 (2896 to 3664) | 1764 (1475 to 2045) | 997 (789 to 1194) |
| **Male under 75** | 1340 (1103 to 1583) | 2671 (2255 to 3065) | 1842 (1548 to 2137) | 2479 (2182 to 2765) | 1358 (1128 to 1578) | 735 (581 to 881) |
| **Female under 75** | 363 (288 to 441) | 933 (776 to 1077) | 550 (459 to 640) | 806 (699 to 910) | 406 (337 to 475) | 262 (205 to 317) |
| **Variant 2 : mean differences in the fourth quartile of difference in**  **FSAm-NPS** | | | | | |  |
| **Total** | -1414 (-2404 to -450) | -1342 (-1820 to -859) | -983 (-1799 to -224) | 1808 (1143 to 2446) | -2186 (-3131 to -1302) | -7389 (-9755 to -5237) |
| **Under 75** | -249 (-586 to 102) | -432 (-608 to -253) | -91 (-305 to 108) | 512 (370 to 657) | -497 (-728 to -273) | -2165 (-2752 to -1623) |
| **Male under 75** | -102 (-348 to 156) | -296 (-420 to -171) | -91 (-255 to 61) | 402 (292 to 515) | -392 (-570 to -220) | -1685 (-2141 to -1269) |
| **Female under 75** | -146 (-244 to -43) | -136 (-191 to -82) | 0 (-52 to 48) | 110 (71 to 149) | -105 (-163 to -48) | -481 (-615 to -356) |

MTL: Multiple Traffic Lights; HSR: Health Star Rating; RIs: Reference Intakes.

# Table S4. Number of deaths avoided through the use of FoPLs (and control situation) using differences by sex

|  | **MTL** | **HSR** | **RIs** | **Nutri-Score** | **SENS** | **Control** |
| --- | --- | --- | --- | --- | --- | --- |
| **Mean differences observed among men** | | | | | | |
| **Total** | 3827 (2596 to 5087) | 7321 (5749 to 8875) | 4605 (3822 to 5360) | 7280 (6298 to 8210) | 656 (10 to 1284) | 180 (-205 to 528) |
| **Under 75** | 1074 (792 to 1380) | 2182 (1824 to 2546) | 1536 (1312 to 1756) | 2350 (2014 to 2656) | 447 (230 to 653) | 7 (-112 to 115) |
| **Male under 75** | 851 (638 to 1084) | 1655 (1381 to 1933) | 1173 (1004 to 1342) | 1739 (1494 to 1962) | 350 (179 to 512) | -11 (-103 to 76) |
| **Female under 75** | 223 (152 to 299) | 527 (436 to 617) | 363 (305 to 419) | 611 (512 to 702) | 97 (42 to 151) | 15 (-10 to 40) |
| **Mean differences observed among women** | | | | | | |
| **Total** | 3505 (2654 to 4400) | 5965 (4870 to 7077) | 4102 (3443 to 4794) | 7765 (6657 to 8837) | 2666 (2034 to 3266) | -441 (-1035 to 86) |
| **Under 75** | 1271 (1043 to 1501) | 1947 (1647 to 2236) | 1367 (1153 to 1580) | 2524 (2215 to 2824) | 976 (787 to 1150) | -89 (-244 to 51) |
| **Male under 75** | 1007 (833 to 1185) | 1456 (1225 to 1684) | 1014 (855 to 1173) | 1906 (1675 to 2132) | 731 (578 to 870) | -85 (-203 to 22) |
| **Female under 75** | 264 (210 to 319) | 491 (410 to 570) | 353 (292 to 412) | 618 (533 to 698) | 245 (195 to 295) | -4 (-41 to 32) |

1. Crosetto P, Lacroix A, Muller L, Ruffieux B. Modification des achats alimentaires en réponse à cinq logos nutritionnels. Cah Nutr Diététique. 1 juin 2017;52(3):129‑33.

2. Crosetto P, Lacroix A, Muller L, Ruffieux B. Nutritional and economic impact of 5 alternative front-of-pack nutritional labels: experimental evidence. Work Pap GAEL N° 112018. 2018;

3. EUR-Lex - 32011R1169 - EN - EUR-Lex [Internet]. [cité 19 janv 2018]. Disponible sur: http://eur-lex.europa.eu/legal-content/EN/ALL/?uri=CELEX%3A32011R1169

4. Food Standard Agency. Front-of-pack Traffic light signpost labelling Technical Guidance; Food Standard Agency: Kingsway, UK. 2007 p.

5. Food and Drink Federation. Reference Intakes (previously Guideline Daily Amounts). 3 juill 2017;

6. Haut Conseil de la santé publique. Avis relatif à l’information sur la qualité nutritionnelle des produits alimentaires [Internet]. 2015 juin p. Disponible sur: http://www.hcsp.fr/explore.cgi/avisrapportsdomaine?clefr=519

7. Communiqué de presse. Marisol Touraine se félicite des résultats des études sur l’impact d’un logo nutritionnel : leur intérêt et l’efficacité du logo Nutri-score sont démontrés. Paris, Ministère des Affaires sociales et de la Santé. 2017 mars p.

8. Health AGD of. Governance [Internet]. Australian Government Department of Health; [cité 11 janv 2018]. Disponible sur: http://healthstarrating.gov.au/internet/healthstarrating/publishing.nsf/content/Governance

9. Darmon N, Maillot M, Braesco V, Tafournel E. L’Algorithme du Système d’Etiquetage Nutritionnel Simplifié (SENS). Développement, description et validation. Avalaible online: http://www.nutrition-quantitative.com/Rapport_SENS.pdf(accessed on 10 August 2017). 2015 p.

10. Rayner M, Scarborough P, Lobstein T. The UK Ofcom Nutrient Profiling Model - Defining « Healthy » and « Unhealthy » Food and Drinks for TV Advertising to Children. Available online: https://www.ndph.ox.ac.uk/cpnp/files/about/uk-ofcom-nutrient-profile-model.pdf (accessed on 10 August 2017). 2009 p.

11. Organisation for Economic Co-operation and Development. The OECD list of social indicators. Paris: Organisation for Economic Co-operation and Development; 1982. (OECD social indicator development programme ; 5.).

12. Hercberg S, Castetbon K, Czernichow S, Malon A, Mejean C, Kesse E, et al. The Nutrinet-Sante Study: a web-based prospective study on the relationship between nutrition and health and determinants of dietary patterns and nutritional status. BMCPublic Health. 11 mai 2010;10:242-.

13. IPAQ Group. Guidelines for Data Processing and Analyses of the International Physical Activity Questionnaire (IPAQ). 2005 p.

14. Les résultats des recensements de la population | Insee [Internet]. [cité 10 janv 2018]. Disponible sur: https://www.insee.fr/fr/information/2008354

15. Lassale C, Peneau S, Touvier M, Julia C, Galan P, Hercberg S, et al. Validity of web-based self-reported weight and height: results of the Nutrinet-Sante study. JMedInternetRes. 8 août 2013;15:e152-.

16. Lassale C, Castetbon K, Laporte F, Deschamps V, Vernay M, Camilleri GM, et al. Correlations between Fruit, Vegetables, Fish, Vitamins, and Fatty Acids Estimated by Web-Based Nonconsecutive Dietary Records and Respective Biomarkers of Nutritional Status. JAcadNutrDiet. mars 2016;116:427‑38.

17. Touvier M, Mejean C, Kesse-Guyot E, Pollet C, Malon A, Castetbon K, et al. Comparison between web-based and paper versions of a self-administered anthropometric questionnaire. EurJEpidemiol. mai 2010;25:287‑96.

18. Le Moullec N, Deheeger M, Preziosi P, Montero P, Valeix P, Rolland-Cachera M. Validation du manuel photo utilisé pour l’enquête alimentaire de l’étude SU.VI.MAX. [Validation of the food portion size booklet used in the SU.VI.MAX study] (in French). Cah Nutr Diet. 1996;

19. Arnault N, Caillot L, Castetbon K, Coronel S, Deschamps V, Fezeu L. Table de composition des aliments, étude NutriNet-Santé. [Food composition table, NutriNet-Santé study] (in French). Paris Éditions Inser. 2013;

20. Black AE. Critical evaluation of energy intake using the Goldberg cut-off for energy intake:basal metabolic rate. A practical guide to its calculation, use and limitations. IntJObesRelat Metab Disord. sept 2000;24:1119‑30.

21. Centre d’épidémiologie sur les causes médicales de décès (CépiDC). CIM-10 Version:2008 [Internet]. [cité 10 janv 2018]. Disponible sur: http://apps.who.int/classifications/icd10/browse/2008/fr

22. Évolution et structure de la population en 2014 | Insee [Internet]. [cité 10 janv 2018]. Disponible sur: https://www.insee.fr/fr/statistiques/2862200#consulter

23. Scarborough P, Harrington RA, Mizdrak A, Zhou LM, Doherty A. The Preventable Risk Integrated ModEl and Its Use to Estimate the Health Impact of Public Health Policy Scenarios. Scientifica(Cairo). 2014;2014:748750-.
